# NanoSIMS analysis of water content in bridgmanite at the micron scale: An experimental approach to probe water in Earth’s deep mantle

**DOI:** 10.3389/fchem.2023.1166593

**Published:** 2023-04-07

**Authors:** Ya-Nan Yang, Zhixue Du, Wenhua Lu, Yue Qi, Yan-Qiang Zhang, Wan-Feng Zhang, Peng-Fei Zhang

**Affiliations:** ^1^ State Key Laboratory of Isotope Geochemistry, Guangzhou Institute of Geochemistry, Chinese Academy of Sciences, Guangzhou, China; ^2^ CAS Center for Excellence in Deep Earth Science, Guangzhou, China; ^3^ College of Earth and Planetary Sciences, University of Chinese Academy of Sciences, Beijing, China; ^4^ Faculty of Earth Resources, China University of Geosciences, Wuhan, China

**Keywords:** water, bridgmanite, NanoSIMS, high pressure, deep Earth

## Abstract

Water, in trace amounts, can greatly alter chemical and physical properties of mantle minerals and exert primary control on Earth’s dynamics. Quantifying how water is retained and distributed in Earth’s deep interior is essential to our understanding of Earth’s origin and evolution. While directly sampling Earth’s deep interior remains challenging, the experimental technique using laser-heated diamond anvil cell (LH-DAC) is likely the only method available to synthesize and recover analog specimens throughout Earth’s lower mantle conditions. The recovered samples, however, are typically of micron sizes and require high spatial resolution to analyze their water abundance. Here we use nano-scale secondary ion mass spectrometry (NanoSIMS) to characterize water content in bridgmanite, the most abundant mineral in Earth’s lower mantle. We have established two working standards of natural orthopyroxene that are likely suitable for calibrating water concentration in bridgmanite, *i.e.*, A119(H_2_O) = 99 ± 13 μg/g (1SD) and A158(H_2_O) = 293 ± 23 μg/g (1SD). We find that matrix effect among orthopyroxene, olivine, and glass is less than 10%, while that between orthopyroxene and clinopyroxene can be up to 20%. Using our calibration, a bridgmanite synthesized by LH-DAC at 33 ± 1 GPa and 3,690 ± 120 K is measured to contain 1,099 ± 14 μg/g water, with partition coefficient of water between bridgmanite and silicate melt ∼0.025, providing the first measurement at such condition. Applying the unique analytical capability of NanoSIMS to minute samples recovered from LH-DAC opens a new window to probe water and other volatiles in Earth’s deep mantle.

## Introduction

**FIGURE 1:**
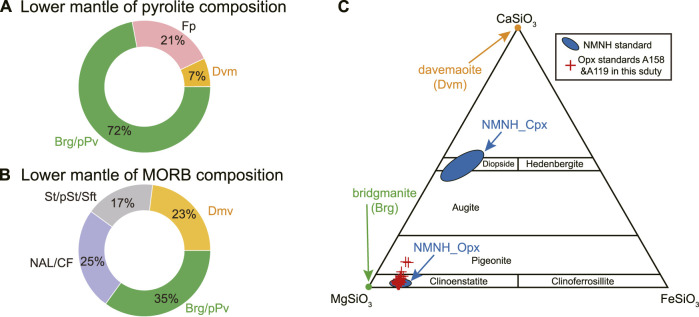
Mineralogy of lower mantle of two different bulk compositions, *i.e.*, pyrolite **(A)** and mid-ocean ridge basalt (MORB), **(B)**, with the weight proportion of main phases indicated. The phase proportion is taken from work by Hirose et al. (2005) and Murakami et al. (2005). Brg, bridgmanite. pPv, post-perovskite. Dvm, davemaoite. Fp, ferropericlase. St, stishovite. pSt, post-stishovite. Sft, seifertite. NAL, new hexagonal aluminous phase. CF, calcium-ferrite phase. **(C)** Chemical composition comparison of bridgmanite and davamaoite relative to the NMNH clinopyroxene (Cpx) and orthopyroxene (Opx) water abundance standards (Kumamoto et al., 2017). NMNH represents the National Museum of Natural Hisynthetic fused silica with certifiedstory. Also shown are Opx standards A158 and A119 established in this study.

Water is a key gradient that makes our planet Earth habitable. The ocean on the surface is the largest known reservoir of water, but even larger ones likely reside within the deep Earth (Keppler, 2014; Li Y et al. 2020; Pearson et al., 2014; Tagawa et al., 2021). Although dissolved in mantle minerals in trace amounts, water can greatly influence their phase relations, transport, and elastic properties (e.g., Inoue, 1994; Du et al., 2019; Freitas and Manthilake, 2019; Zhang et al., 2022). Despite much progress on how water may be dissolved in upper mantle and transition zone minerals (e.g., Hauri et al., 2006; Inoue et al., 2010; Pearson et al., 2014; Gu et al., 2022), its quantity and chemical properties throughout lower mantle are largely unconstrained (e.g., Litasov et al., 2003; Fu et al., 2019; Lin et al., 2020; Ishii et al., 2022a). Since Bridgmanite (Brg) is the most abundant mineral in silicate mantle (Figures 1A,B, e.g., Hirose et al., 2017; Irifune and Tsuchiya., 2015; Marquardt and Thomson., 2020), its water content would provide critical constraints on how water is distributed and cycled within the Earth. Due to rare occurrence of natural bridgmanite, laser-heated diamond anvil cell (LH-DAC), which routinely generates pressure and temperature conditions throughout Earth’s low mantle, could be used to synthesize analog samples (e.g., Akahama Kawamura, 2006; Du et al., 2013; Anzellini and Boccato., 2020). Yet the recovered bridgmanite samples are of usually 1–3 μm sizes (Fischer et al., 2020; Nabiei et al., 2021; Blanchard et al., 2022a; Baron et al., 2022), which presents an immense challenge to quantify their water contents.

Fourier transform infrared reflection (FTIR) spectroscopy and secondary ion mass spectrometry (SIMS) are commonly used to quantify water contents in nominally anhydrous minerals (NAMs). FTIR spectroscopy is advantageous to acquire both details of water speciation and total water contents (Stolper, 1982; Mercier et al., 2010; Yasuda, 2014). However, FTIR typically requires a double-polished specimen with a spatial resolution of tens of microns, which nearly precludes its use for LH-DAC samples. Complex and ambiguous baseline corrections may also undermine the accuracy of FTIR results (Mosenfelder and Rossman, 2013a; Mosenfelder and Rossman, 2013b). On the other hand, sample preparation for SIMS analysis is more straightforward, where single-side sample polishing is sufficient (Hauri et al., 2002; Koga et al., 2003). More importantly, SIMS excels at its ultra-high spatial resolution on the level of micron-submicron (Saal et al., 2008; Hauri et al., 2017; Hu et al., 2021; Li et al., 2021). With the latest nano-scale SIMS NanoSIMS 50L, an additional advantage is its capability to measure up to seven masses simultaneously without switching magnetic field, thereby reducing analytical uncertainties as repeatability is greatly improved (Hauri et al., 2011; Steinhauser et al., 2012). Moreover, the simultaneous collection of multiple elements increases overall throughput, which is critical for a micron-size target that does not tolerate long-period sample consumption. Therefore, NanoSIMS is an ideal technique to measure water abundance in micron-size bridgmanite and other phases recovered by LH-DAC (Badro et al., 2007; Suer et al., 2017; Fischer et al., 2020; Suer et al., 2021; Blanchard et al., 2022b).

It is desirable that NanoSIMS reference materials match as close as possible to the chemical and physical matrix of the target sample for quantitative analysis. Unfortunately, reference bridgmanite is not currently available for calibrating water contents by SIMS, where either hornblende (Murakami et al., 2002; Litasov et al., 2003; Inoue et al., 2010) or basaltic glass (Fu et al., 2019) were used previously. Orthopyroxene (Opx) is chemically more similar to Bridgmanite (MgSiO_3_-rich perovskite, Figure 1C). Adopting Opx as a reference to calibrate water content in bridgmanite may be more appropriate. Here we present NanoSIMS analysis of water in pyroxene based on a set of reference materials from the Smithsonian Institution. Following previous protocols, we characterize two natural Opx that could serve as in-house working standards to calibrate water abundance in bridgmanite. Water abundance in bridgmanite produced by LH-DAC at 33 ± 1 GPa and 3,690 ± 120 K is measured to illustrate the capability of NanoSIMS imaging analysis.

## Materials and methods

### Sample description

A set of SIMS reference materials for measuring water (H_2_O) in Opx and clinopyroxene (Cpx) was acquired from the Department of Mineral Sciences, Smithsonian Institution (Kumamoto et al., 2017) and cast into two new mounts (Opx S2598; Cpx S2599). A standard mount with Opx and Cpx reference materials (SM3) was used to calibrate the water contents of Opx and Cpx grains in mounts S2598 and S2599 (Table 1).

**TABLE 1:** Reference materials of Cpx and Opx.

Mineral	Sample No	H_2_O (μg/g)^a^	1 σ (μg/g)^a^	Standard mount SM3	New mount	H_2_O (μg/g)^b^	1 σ (μg/g)^b^
Opx	116610-29	62	5	⊕	⊙	55	3
	116610-18	119	18		⊙	82	6
	116610-10	128	12	⊕			
	117213-5	169	11		⊙	165	13
	118317-2	182	19	⊕			
	117322-245	211	12		⊙	210	11
	116610-21	215	21	⊕			
	116610-15	234	21	⊕			
	116610-16	264	28		⊙	214	9
	116610-5	309	27	⊕	⊙	265	21
	A119				◎	99	13
	A158				◎	293	23
Cpx	118319	5	8		⊙	1.2	1.0
	118318	62	9	⊕			
	117322-242	127	16		⊙	130	5
	116610-18	199	27	⊕			
	117213-5	315	40	⊕	⊙	295	11
	116610-21	354	50	⊕	⊙	326	11
	116610-15	441	61		⊙	451	17
	118316-1	427	59	⊕			
	116610-5	544	79	⊕			

^a^ Reference water contents from Kumamoto et al. (2017).

^b^ Water abundance that is calibrated against the standard mount SM3 (NMNH, catalog number 118331).

⊕ Reference materials in the standard mount SM3.

⊙ Reference material chips in the new standard mounts, *i.e.*, S2598 Opx and S2599 Cpx.

◎ New natural Opx reference materials characterized in this study.

Natural Opx grains (A119 and A158) were separated from two harzburgite samples from the Acoje block of the Zambales ophiolite, Philippines (Zhang P et al., 2020; Zhang et al., 2021) to evaluate the homogeneity of water contents and their potential to serve as in-house working standards. Both harzburgite samples show porphyroblastic texture with Opx as the porphyroblasts and have been moderately serpentinized. Sample A158 has ∼5 modal% Cpx, defining it as Cpx-rich harzburgite. By contrast, there is only ∼2 modal% Cpx in sample A119, which is classified as Cpx-poor harzburgite. The chemical compositions of both samples are similar to those of Opx reference materials from the Smithsonian Institution (Figure 1C and Supplementary Table S2).

References for olivine (KLB-1, ICH-30, Mongok, San Carlos; Zhang W et al., 2020) and silicate glass (EPR-G3, IND-G1, IND-G2, FG-G2, Shimizu et al., 2017; ALV519-4-1; Helo et al., 2011) were also measured to evaluate matrix effect during NanoSIMS analysis. Suprasil 3002 is a high-purity synthetic fused silica with certified OH contents of 1 μg/g manufactured by flame hydrolysis by Heraeus. Both carbon (C) and H_2_O contents of the Suprasil 3002 had been demonstrated to be very low (0.068 ± 0.043 μg/g of C and 0.99 ± 0.36 μg/g of H_2_O, Wetzel et al., 2015). The Suprasil 3002 glass is measured in this study to have 0.54 ± 0.03 μg/g of H_2_O, 0.052 ± 0.003 μg/g of CO_2_, 0.029 ± 0.004 μg/g of F, 0.0004 ± 0.0001 μg/g of P, and 0.003 ± 0.001 μg/g of S (1SD, Supplementary Table S3). Therefore, the Suprasil 3002 glass is adopted to assess the background level of C, H_2_O, F, P, and S. Chlorine (Cl) contents of Suprasil 3002 glass produced by Heraeus could reach 1,000 μg/g - 3,000 μg/g (976 ± 45 μg/g in this study, 1SD, Supplementary Table S3), excluding its use to monitor the chlorine background. We, therefore, utilize San Carlos olivine (Ol) to assess Cl background, which has a very low abundance of Cl down to 0.07 ± 0.01 μg/g (Supplementary Table S3).

LH-DAC sample was prepared at the State Key Laboratory of Isotope Geochemistry, Guangzhou Institute of Geochemistry, Chinese Academy of Sciences (GIGCAS). LH-DAC experiment was performed by using diamond anvils with 300 μm diameter culet and rhenium (Re) gasket. Re gasket was pre-indented to ∼40 μm in thickness and laser-drilled with a ∼100 μm diameter hole to sever as the sample chamber (Supplementary Figure S1). An F- and Cl-bearing silicate glass with a composition modified from pyrolite (MP glass, Supplementary Table S1) was synthesized using a piston cylinder. We loaded two MP glass disks sandwiching a FeS-dopped silicate powder layer into the chamber of the Re gasket (Supplementary Figure S1). The loaded sample was dampened by pure alcohol (99.99%) to add H and C before compressing to target pressure. The sample was heated by a defocused 1,064 nm laser on both sides of diamond anvil cells, at peak power for 1 s. Pressures were determined using the Raman shift of the diamond before and after the heating (Akahama and Kawamura., 2006). Temperatures from both sides were determined by a two-dimensional temperature mapping system similar to that of Du et al. (2013). Temperature fluctuations during the heating at peak laser power are within 50 K. After quenching and decompression to ambient conditions, recovered samples along with the Re gasket were mounted in epoxy and hand-polished perpendicular to the compressional axis using Al_2_O_3_-coated sandpapers. Subsequently, polished samples were taken out of the epoxy and held by a metal holder for *in situ* NanoSIMS analyses.

It is critical to achieving a low level of background for accurate analysis of volatile contents in NAMs (Koga et al., 2003; Zhang et al., 2018). We used tin-based alloy instead of epoxy to mount our reference materials (Zhang et al., 2018) and kept the sample chamber at ultrahigh vacuum status (below 5 × 10^−10^ mbar) throughout measurements.

### Analytical settings

**FIGURE 2:**
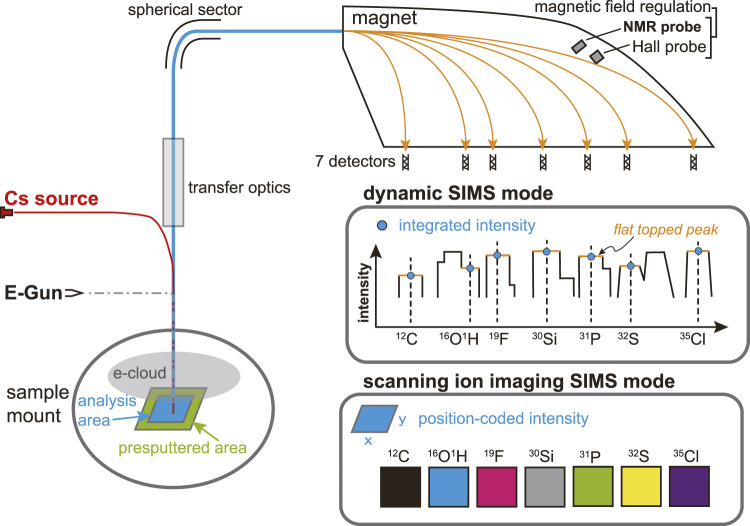
Illustration of the two modes of NanoSIMS multi-collection analysis used in this study: dynamic SIMS mode and scanning ion imaging SIMS mode.

CAMECA NanoSIMS 50 L ion microprobe at GIGCAS was used in this study. A cesium (Cs^+^) primary beam was rastered over the sample surface with an impact energy of 16 keV to yield secondary negative ions. Given our silicate sample being an insulator, the electron gun was applied to generate electrons to hover above the sample surface to compensate potential charging effect (e-cloud, Figure 2). Secondary ions ^12^C^−^, ^16^O^1^H^−^, ^19^F^−^, ^30^Si^−^, ^31^P^−^, ^32^S^−^, and ^35^Cl^−^ are measured simultaneously within the 7 electron multipliers in multi-collection detection mode. High mass resolution is required to separate potential isotopic interferences on mass peaks of interest. For example, to separate ^16^O^1^H^−^ from closest interference ^17^O^−^, the mass resolution should reach at least 5000. Entrance slit 4 (ES4 width 20 μm) together with aperture slit 2 (AS2 width 200 μm) were used to achieve the required mass resolution. Both Hall and nuclear magnetic resonance (NMR) probes were activated to regulate the magnetic field for higher stability. This could avoid the side effect of varying stray magnetic fields on the electron beam when switching the B-field of the instrument magnet (Mosenfelder et al., 2011). The ^16^O^1^H^−^ instead of ^1^H^−^ was chosen as the species to estimate water content in this study due to its relatively higher ionization efficiency, easiness of simultaneous collection with other six mass species (*i.e.,* C, F, Si, P, S, and Cl) without switching magnetic field, and less tendency of being affected by the stray magnetic field.

**FIGURE 3:**
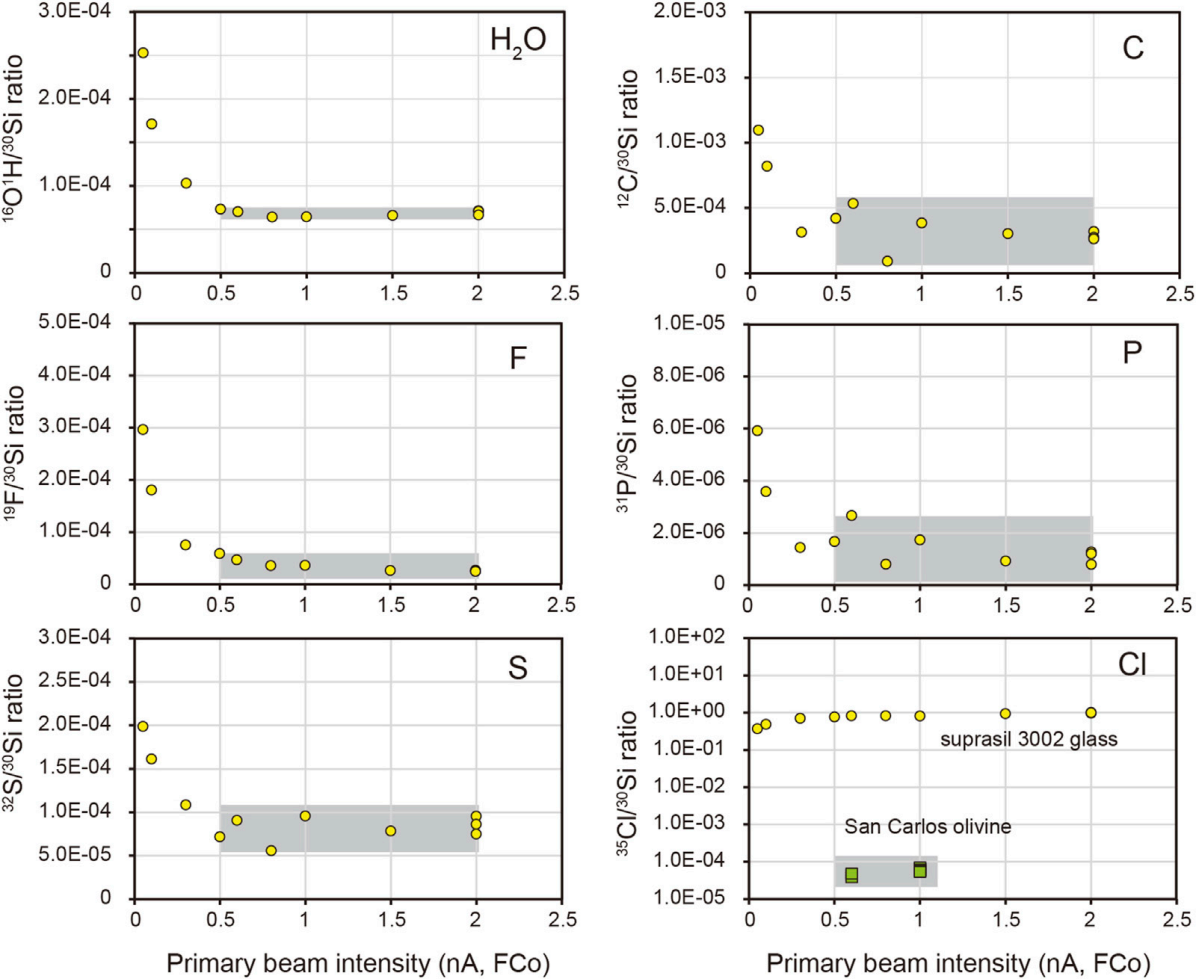
The ^30^Si-normalized ratios with varying primary beam intensity on Suprasil 3002 glass (yellow circle) and San Carlos olivine (green square). The grey area indicates the range of primary beam intensity that is optimal for reducing the background.

High primary beam intensity could significantly reduce the background signal such as contamination from the sample surface (Stephant et al., 2014; Hu et al., 2015; Li R et al., 2022). Varying primary currents were applied in this study to determine the optimal primary beam intensity for volatile analysis using Suprasil 3002 glass and San Carlos olivine. It is shown that the ratios of OH/Si, C/Si, F/Si, P/Si, and S/Si decrease rapidly when increasing the primary ion intensity from 0.05 nA to 0.5 nA and are stable even when the primary ion intensity is elevated up to 2 nA (Figure 3). Therefore, a primary beam intensity of at least 0.5 nA during analysis was used to reduce the background.

Two different modes to collect secondary ions are available, *i.e.*, dynamic SIMS mode and scanning ion imaging SIMS mode (Figure 2). In dynamic SIMS mode (*i.e.*, isotope analysis as defined by CAMECA), a high current (2–7 nA) of Cs^+^ primary beam is set to presputter an area of 30 μm × 30 μm for 240 s on the sample surface, after which the primary beam current is switched to 1 nA to analyze the central area of 10 μm × 10 μm (64 × 64 pixels) using the beam blanking option. The counting time for each scanning frame is 1 s. The secondary ion signal is integrated to yield a total intensity from the analysis area for each mass. Using dynamic SIMS mode, we recalibrated water contents of Cpx and Opx minerals from the Smithsonian Institution and natural Opx grains (A119 and A158). On the other hand, in scanning ion imaging SIMS mode, the instrument setting is similar to those of the dynamic SIMS mode but the secondary ion intensity is position-coded as scanning secondary ion images of varying size according to the target sample with 256 × 256 pixels. When analyzing DAC samples by scanning ion imaging SIMS mode, the primary beam current during analysis is changed to 0.5 nA for high spatial resolution. The counting time for each scanning frame is 16 s. Measurements of unknown samples are always accompanied by concurrent analyses of reference materials using the same analysis recipe, either in dynamic SIMS mode or scanning ion imaging SIMS mode.

### Data processing method

The first step to process the NanoSIMS data in this study is to filter out data by comparing the error predicted by the Poisson counting statistics (σ_Poisson_) to the standard error for individual analysis (σ_mean_). Data with σ_mean_/σ_Poisson_ > 5 are not used (Mosenfelder et al., 2011). Then several analyses for one sample can be evaluated with attention to abnormal ^12^C/^30^Si or ^19^F/^30^Si ratios that may indicate potential contamination on sample surfaces or measurement overlapping inclusions of hydrous phases. The background is subtracted by those of Suprasil 3002 glass for C, H_2_O, F, P, S and San Carlos olivine for Cl. More than two analyses were done for each standard, from which an average ^30^Si-normalized ratio and its standard deviation were derived. The calibration curve was constructed by a weighted least-squares linear regression and forced through the origin following the method by Kumamoto et al. (2017).

## Results

### Calibration for new standard mounts

**FIGURE 4:**
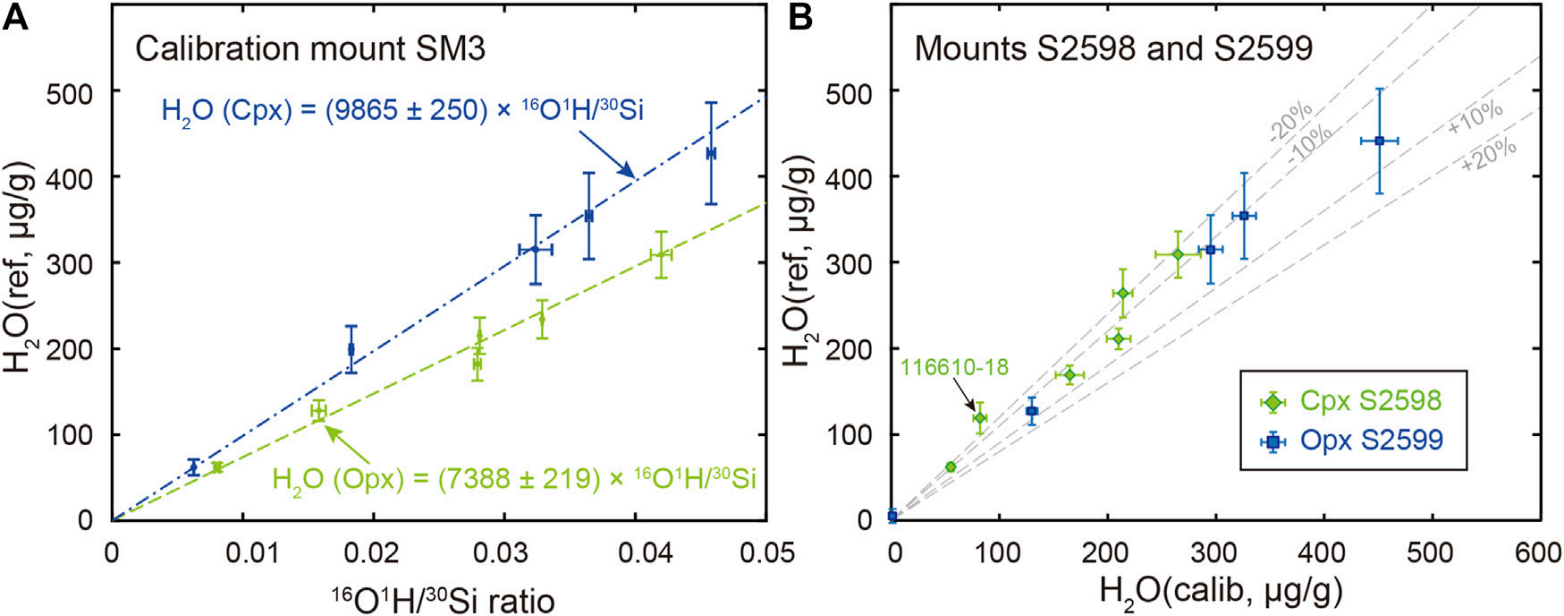
**(A)** Water content calibration curves constructed by Opx (green dash line) and Cpx (blue dash-dotted line) on standard mount SM3 as specified in Table 1 **(B)** Comparison of calibrated water contents (*i.e.*, H_2_O calib) for newly-requested Opx (S2598, green diamonds) and Cpx (S2599, blue squares) with their reference values (i.e., H_2_O ref). Percent deviation of calibrated water abundance from reference values (±10% and ±20%) are indicated as grey dashed lines. Negative deviation (−10% and −20%) indicates calibrated water contents are lower than that of reference value, while positive deviation (+10% and +20%) indicates calibrated water contents are higher than that of the reference value.

The water abundance of Opx and Cpx chips acquired from the Smithsonian Institution (new standard mounts Opx S2598 and Cpx S2599) is recalibrated against the standard mount SM3 (Figure 4A) due to potential intergranular variations (Kumamoto et al., 2017). Except for the Opx 116610-18 which has relatively low water contents, all other calibrated water contents for new-requested reference chips fall within ±20% deviation from reference water contents (Figure 4B, Supplementary Table S4).

### New natural Opx working standards

**FIGURE 5:**
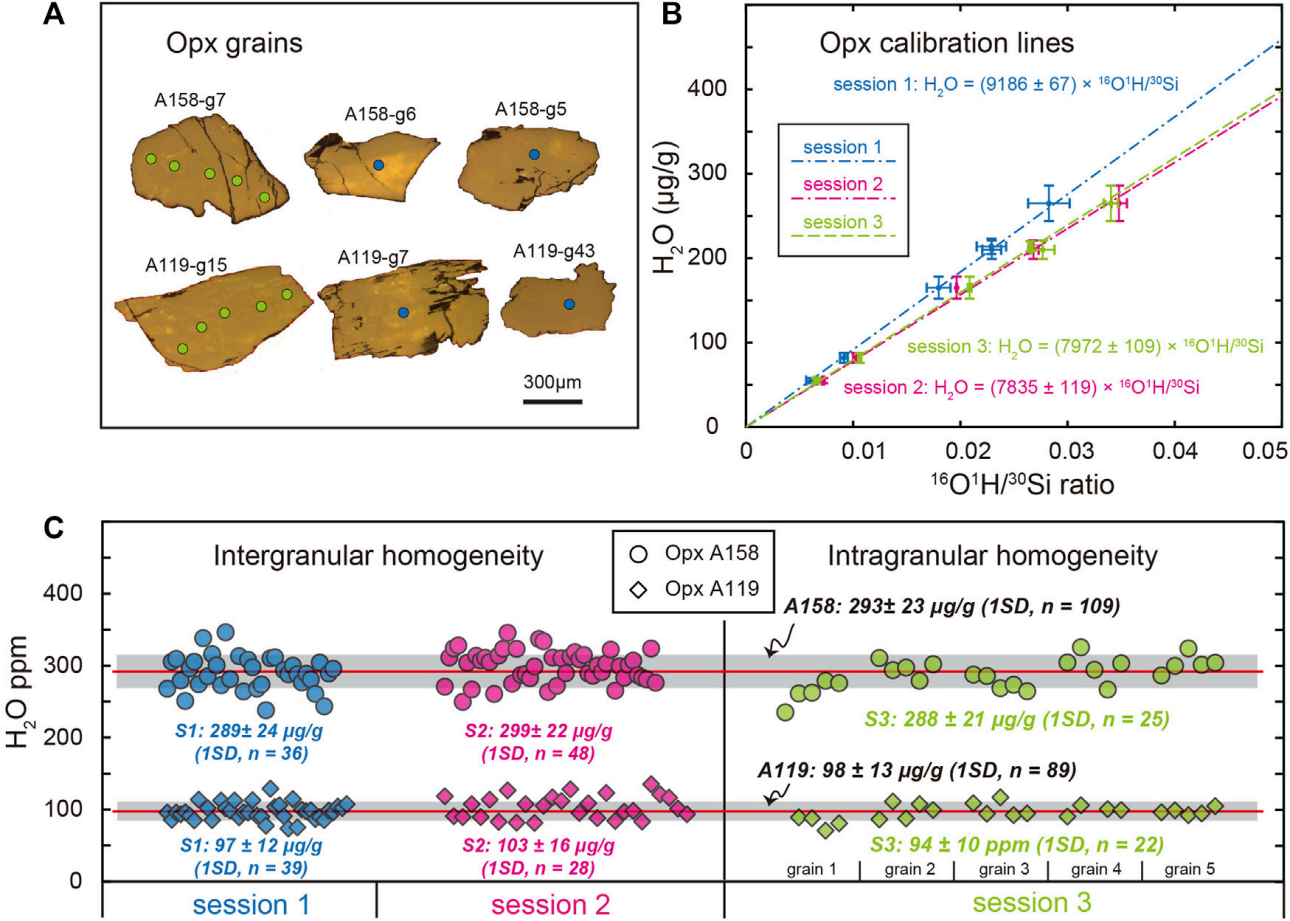
Characterizations of water abundance in A158 and A119 Opx grains in three analytical sessions. **(A)** Representative reflection photos of A158 and A119 Opx grains with grain number indicated. Blue and green circles on the Opx grains indicate analysis spots during sessions 1 and 3, respectively. **(B)** The Opx H_2_O calibration lines for session 1 (blue dash-dotted line, primary beam current 0.5 nA), session 2 (magenta dash-dash-dotted line, primary beam current 1.0 nA), and session 3 (green dash line, primary beam current 1.0 nA). **(C)** Intergranular and intragranular homogeneity of water abundance in Opx A158 (circle) and A119 (diamond). Blue, magenta, and green refer to data obtained in sessions 1, 2, and 3, respectively. The integrated mean water contents of all Opx A158 and A119 are also shown, for which the horizontal red line represents the mean value and the grey area indicates ± 1SD range.

The water abundance of Opx separates A158 and A119 was measured to assess their intergranular and intragranular homogeneity during sessions 1 and 2, and session 3, respectively (Figure 5, Supplementary Table S5). In sessions 1 and 2, the analysis spots are mainly at the center domain of each Opx grain, while spot analyses in session 3 are along profiles across representative Opx grains (Figure 5A). Apart from three outliers that either was on grains with cracks or those with abnormal analytical error, 36 and 48 analyses on fifty-one A158 Opx grains in session 1 and 2 yield average H_2_O concentration of 289 ± 24 μg/g (1SD, n = 36) and 299 ± 22 μg/g (1SD, n = 48), respectively (Figure 5C). The intragranular homogeneity test of 5 representative grains for A158 Opx with 4-5 analyses on each grain reveals less than 8% variation of water contents. The 25 measurements of A158 in session 3 give H_2_O concentration of 288 ± 21 μg/g (1SD), which is consistent within analytical uncertainties with the intergranular results. The pooled average H_2_O abundance of A158 Opx in all three sessions is 293 ± 23 μg/g (1SD, n = 109, Figure 5C). As for A119 Opx, average H_2_O abundance of 97 ± 11 μg/g (1SD, n = 39) and 103 ± 16 μg/g (1SD, n = 28) are obtained for A119 Opx grains in sessions 1 and 2, respectively (Figure 5C). Variation of water abundance of 5 representative A119 Opx grains ranges from 4 up to 11% on single grain, which is larger than that for A158 Opx. Twenty-two measurements of A119 Opx result in an average H_2_O concentration of 94 ± 10 μg/g (1SD). The pooled average H_2_O abundance of A119 Opx in all three sessions is 98 ± 13 μg/g (1SD, n = 89, Figure 5C).

### Imaging a LH-DAC sample

**FIGURE 6:**
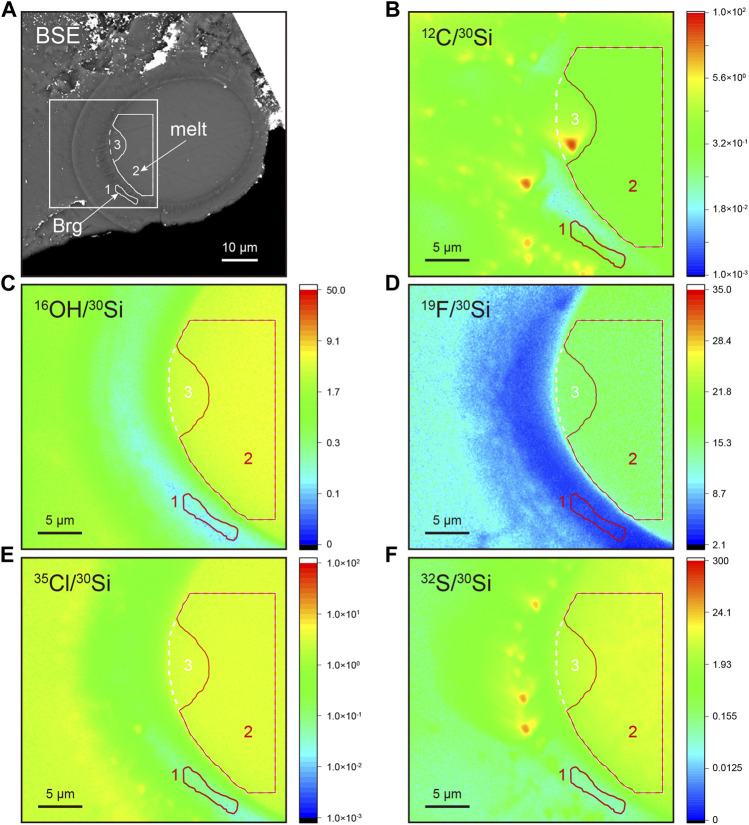
NanoSIMS imaging analysis of bridgmanite synthesized by laser-heated anvil cell at ∼33 GPa and ∼3,690 K **(A)** Backscattered image. Brg, bridgmanite. **(B–F)** NanoSIMS scanning images of ^12^C/^30^Si, ^16^O^1^H/^30^Si, ^19^F/^30^Si, ^35^Cl/^30^Si, and ^32^S/^30^Si ratios. The white square within **(A)** indicates the NanoSIMS scanning area of 30 μm × 30 μm. Closed areas with numbers represent regions of interest within bridgmanite (1) and melt (2, 3). In B-F, ROI#1 and #2 are in red, while ROI#3 is in white. ROI#3 includes ROI#2 except that ROI#3 contains an additional area that shows a relatively high ^12^C/^30^Si ratio.

The LH-DAC sample was recovered from 33 ± 1 GPa and 3,690 ± 120 K to simulate high pressure and high temperature conditions in deep Earth. The exposed area represents a melt ellipsoid rimmed by a thin layer of Brg which crystallizes from the melt phase (Figure 6A, Supplementary Table S6). Regions of interest (ROIs) within melt and Brg are selected according to backscattered electron image (BSE) and NanoSIMS scanning images of ^30^Si-normalized ratios. We select ROIs where ^30^Si-normalized ratios are homogeneous and away from any obvious cracks. The Brg along the margin of the central melt is only about 2 μm in width (Figure 6). The calibrated H_2_O content of Brg is 1,099 ± 14 μg/g (ROI#1, Supplementary Table S6). Carbon is usually used as an indicator of surface contamination. This is clearly shown by the relatively higher ^12^C/^30^Si ratio, as exemplified by the “hotspot” with an extreme ^12^C/^30^Si ratio (Figure 6B). Two ROIs (#2 and #3) are selected within melt to evaluate the potential effect of surface carbon contamination on water abundance. ROI#2 represents a large and homogeneous area within the melt, while ROI#3 includes ROI#2 except that ROI#3 contains an additional area that shows a relatively high ^12^C/^30^Si ratio (Figure 6B). The ^12^C/^30^Si of ROI #3 melt is about 3 times that of ROI #2 (0.910 of #3 vs. 0.314 of #2, Supplementary Table S6). However, the calibrated water abundance of ROIs #2 and 3 melt are 44,226 ± 834 μg/g and 43,083 ± 812 μg/g, respectively, which agree well with each other within analytical uncertainties. Contribution from surface contamination to water abundance of ROI #3 melt in this case, if exists, should be limited. We adopt the H_2_O abundance in the ROI#2 melt to calculate the water partition coefficient (*D*_H2O_), which defines the ratio of the H_2_O concentration in Brg to that in the melt, of about 0.0248 ± 0.0006 (Table 2). Although Opx reference materials are lacking to calibrate fluorine (F), sulfur (S), and chlorine (Cl) abundance in Brg, first-order estimates of the partition coefficient between Brg and melt can be estimated neglecting the matrix effect between Brg and melt during NanoSIMS analysis. Therefore, partition coefficients of F, S, and Cl between Brg and melt are calculated by Si-normalized secondary ion ratios of ROI#1 Brg and ROI#2 melt: *D*_F_ = 0.1783 ± 0.0006, *D*_S_ = 0.0126 ± 0.0001, and *D*_Cl_ = 0.0202 ± 0.0002 (Table 2). The C partition coefficient between Brg and melt is not estimated due to potential surficial carbon contamination.

**TABLE 2:** Estimated partition coefficients of H_2_O, F, S, and Cl between Brg and melt at 33 ± 1 GPa and 3,690 ± 120.

*D*_H2O_^*^	*D*_F_^&^	*D*_S_^&^	*D*_Cl_^&^
0.0248 ± 0.0006	0.1783 ± 0.0006	0.0126 ± 0.0001	0.0202 ± 0.0002

*D_H2O_ is derived from calibrated water abundance in ROI#1 Brg and ROI#2 melt.

&*D*_F_, *D*_S_, and *D*_Cl_, are derived from Si-normalized secondary ion ratios in ROI#1 Brg and ROI#2 melt.

## Discussion

### Reference materials

Reference materials are the basis for quantitative SIMS analysis due to potential isotopic and elemental fractionation mainly during sample sputtering. Although the importance of water abundance within NAMs (e.g., Ol, Cpx, Opx) has long been recognized (Bell and Rossman., 1992; Hirschmann et al., 2005; Rossman, 2006; Aubaud et al., 2007; Peslier, 2010; Mosenfelder et al., 2011; Mosenfelder and Rossman, 2013a; Mosenfelder and Rossman, 2013b), universally-distributed reference materials had not been possible until Kumamoto and collaborators proposed a set of SIMS Cpx and Opx reference materials for measuring water made available from the Department of Mineral Sciences, Smithsonian Institution (Kumamoto et al., 2017). We recalibrated the water abundance in newly allocated Opx and Cpx chips against the standard mount SM3. The deviation of calibrated Cpx water contents from their reference values falls within ±20% (Figure 4B). In contrast, the Opx reveals a larger deviation from their recommended values, especially for 1166101-18 Opx (Figure 4B). Such deviation is expected considering likely intergranular variation of water content (Kumamoto et al., 2017). However, the calibrated water abundance agrees with the reference value within the analytical uncertainties considered.

The common practice of SIMS analysis is to cast the reference material together with the unknown sample on the same mount, which demands a large quantity of reference materials in stock. To augment the Opx water reference material archive, we evaluated the water contents of two samples from harzburgite samples from the Acoje block of the Zambales ophiolite, Philippines. Both intergranular and intragranular variation of the water abundance in A158 Opx grain is less than 8%. In addition, its water content of 293 ± 23 μg/g is comparable to that of 116610-5, the Opx reference material with the highest water content presented by Kumamoto et al. (2017). Thus, A158 Opx could serve as a potential water abundance calibration standard. On the other hand, although the A119 Opx grain displays a larger water variation of up to 15%, its water content of 99 ± 13 μg/g could offer an optional anchor point on the calibration line (Figure 4). Because we used 10 μm × 10 μm analysis when analyzing water concentration in A158 and A119, these two Opx samples are homogenous in water contents at such spatial scale. Therefore, both A158 and A119 Opx are beneficial candidates for Opx water content calibration reference materials by SIMS. It is desirable to independently determine the water content in Opx A158 and A119 standards. Due to challenges of measuring water content by independent techniques such as FTIR, elastic recoil detection analysis, nuclear reaction analysis, and hydrogen manometry as pointed out by Kumamoto et al. (2017), we followed their proposed protocols to characterize pyroxene reference materials, *i.e.*, only NanoSIMS analysis in this study. The accuracy of the measurements is monitored by Opx standards from the Smithsonian Institution (Figure 5B). In addition, it can be seen in Figure 5C that the calibrated water contents of A158 and A119 from three analytical sessions agree well with each other, although the analysis condition of session 1 is different from those of sessions 2 and 3. This indirectly confirms the validity of our analytical methods and the calibrated water contents of pyroxene crystals from the harzburgite samples. Moreover, although there have been ongoing efforts to characterize new Opx and Cpx water reference materials (*e.g.*, Wenzel et al., 2021; Zhang et al., 2023), much more work is still needed considering the large chemical composition range of pyroxene (Figure 1C). Future work on water abundance reference materials may include potential candidates such as recently characterized natural Cpx reference materials for *in situ* strontium isotopic analysis by laser ablation multicollector inductively coupled plasma mass spectrometry (Zhao et al., 2020) and natural Cpx reference materials for *in situ* trace element abundances by laser ablation inductively coupled plasma mass spectrometry (Li W et al. 2022).

### Matrix effect

**FIGURE 7:**
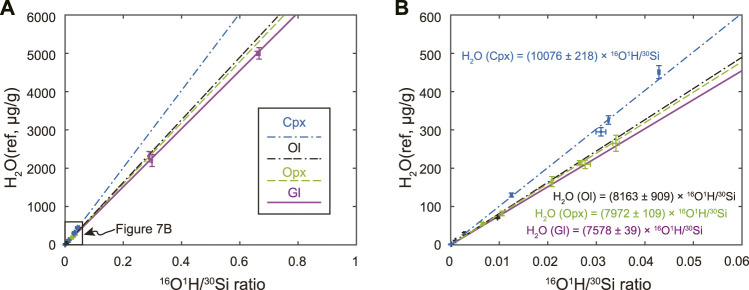
Water abundance calibration curves for clinopyroxene (Cpx, blue dash-dotted line), olivine (Ol, black dash-dash-dotted line), orthopyroxene (Opx, green dash line), and basaltic glass (gl, purple solid line). The right panel **(B)** is an enlarged part of the left panel **(A)** with H_2_O less than 600 μg/g and ^16^O^1^H/^30^Si less than 0.06.

One prominent feature of SIMS analysis is the ionization efficiency of elements/isotopes may differ in target specimens with varying chemical compositions and structures. This results in different minerals displaying contrasting calibration curves for quantitative analysis due to their matrix difference. Figure 7 shows that basaltic glass yields a slightly smaller slope than that of Opx by about 4%. The deviation is about 8% during analysis for the LH-DAC sample (Supplementary Figure S2). This may imply that the difference between the water contents in bridgmanite calibrated in the previous study using basaltic glasses (Fu et al., 2018) from that calibrated using Opx be no larger than 10%. Interestingly, the calibration curve for Ol [Fo = 100 × Mg/(Mg+Fe)] = 89.1 - 99.1, Zhang W et al., 2020), one of the major minerals in the upper mantle, has almost the same slope as for Opx (Figure 7B), which is consistent with the previous study suggesting ≤20% matrix effect for SIMS measurements between Opx and Ol (Mosenfelder and Rossman, 2013a). The similarity matrix effect between Opx and Ol implies that both sets of reference materials can be combined to yield a unified calibration curve with twofold advantages. Such a unified calibration curve not only offers tighter control on the low water abundance of <50 μg/g water for Opx but also extends the accurate estimate of Ol up to water contents of about 300 μg/g (Table 1). The slope for the Cpx calibration curve, however, is about 20% higher than that for Opx likely due to the relative enrichment of CaO in Cpx (Figure 1C; Figure 7B).

Considering the similarity of chemical composition between Opx and Brg (Figure 1C), we tentatively propose to use Opx water reference materials to calibrate water content in Brg for NanoSIMS analysis. However, the matrix effect during SIMS analysis requires reference materials matched to unknown samples both chemically and structurally (*e.g.*, Azevedo-Vannson et al., 2022). Fortunately, both Opx and Brg are orthorhomic (Warren et al., 1930; Liu et al., 1974; Smyth and Swope., 1990; Tschauner et al., 2014). This lends support to validity of using Opx to calibrate water abundance in Brg. Meanwhile, it should be noted the intrinsic structure difference between Opx and Brg, with the former belonging to space group Pbca (Warren et al., 1930; Smyth and Swope., 1990) and the latter Pbnm or Pnma (Liu et al., 1974; Tschauner et al., 2014; Hirose et al., 2017). Future work on synthesizing Brg crystals with homogenous volatile contents is still highly desirable and the effect of structural difference on accurate calibration should be fully explored.

### NanoSIMS analysis of volatile contents in lower mantle minerals

As bridgmanite is the dominant mineral in Earth’s lower mantle, it has been of great interest to investigate the water solubility in Brg and the partitioning between Brg and equilibrated melt using static compression experiments such as LH-DAC. However, water solubility in Brg has not been well-constrained, ranging from less than 10 μg/g to 2,300 μg/g determined either by FTIR or SIMS (Murakami et al., 2002; Bolfan-Casanova et al., 2003; Litasov et al., 2003; Inoue et al., 2010; Fu et al., 2019). Except for the uncertainties from analytical methods, one of the challenges of measuring water contents in Brg is to avoid potential contributions from tiny inclusions of hydrous phases (*e.g.*, Fu et al., 2019; Kaminsky, 2018). This requires *in situ* analytical techniques with high spatial resolution such as NanoSIMS (*e.g.*, Hoppe et al., 2013; Li K et al. 2020; Messenger et al., 2003). It is particularly advantageous when using the scanning ion imaging mode that allows for identifying regions of interest that are clear of any surface contamination, minuscule cracks, or miscellaneous phases that are not readily identified by the BSE image (Figure 6). The water contents in our Brg is 1,099 ± 14 μg/g, which is consistent with the water concentration of 1,020 ± 70 μg/g in high-quality and inclusion-free single-crystal Brg determined by NanoSIMS (Fu et al., 2019), supporting the significant role of (Al,Fe)-bearing Brg in hosting large quantity of water in the lower mantle (*e.g.*, Fu et al., 2019; Litasov et al., 2003; Murakami et al., 2002). Importantly, an additional benefit of using NanoSIMS is that H_2_O, F, S, and Cl abundance in Brg can be determined simultaneously, and their partition coefficients between Brg and equilibrating melt be obtained accordingly (Figure 6; Table 2). Although the detector configuration in this study included ^12^C^−^, ^16^O^1^H^−^, ^19^F^−^, ^30^Si^−^, ^31^P^−^, ^32^S^−^, and ^35^Cl^−^, they can be changed to measure other elements of interest, such as replacing the ^31^P^−^ with ^14^N^16^O^−^ and ^30^Si^−^ with ^29^Si^−^ (Gao et al., 2022), where either ^30^Si or ^29^Si serves as the normalized species. Considering that the partitioning behavior of any of the above-mentioned volatile elements may be affected by others (Iacono-Marziano et al., 2012; Li et al., 2015; Gaillard et al., 2022), simultaneous detection of several volatile elements is beneficial to a comprehensive evaluation of their likely interdependent partitioning behavior. The partition coefficient of volatiles between the melt and crystalline phases (*e.g.*, bridgmanite) is affected by several factors including chemical composition, temperature, pressure, and oxygen fugacity (Litasov et al., 2003; Fu et al., 2019; Liu et al., 2021; Ishii et al., 2022b), which is still poorly constrained. We advocate comprehensive study in the future by LH-DAC-NanoSIMS to improve our understanding of Earth’s deep volatile budget and its chemical evolution.

## Conclusion

Two new natural Opx separates are characterized as potential reference materials for measuring water abundance in bridgmanite. We find that scanning ion imaging SIMS mode is key to determining volatile abundance (*i.e*., C, H_2_O, F, S, Cl) in micron-size crystalline phases synthesized by LH-DAC. Integration of high-spatial-resolution capability offered by NanoSIMS and deep Earth minerals synthesized by high temperature and high pressure apparatus LH-DAC will be powerful to probe water and other volatiles in the deep interior of Earth and other planets. Future works will include finding matrix-matched reference materials for calibrating water contents in davemaoite, ferropericlase, stishovite/post-stishovite/seifertite, and new hexagonal aluminous phase/calcium-ferrite phase, which together with bridgmanite make up the bulk of Earth’s lower mantle.

## Data Availability

The original contributions presented in the study are included in the article/Supplementary Material, further inquiries can be directed to the corresponding authors.
