# Corrigendum: NanoSIMS analysis of water content in bridgmanite at the micron scale: an experimental approach to probe water in Earth’s deep mantle

**DOI:** 10.3389/fchem.2023.1210511

**Published:** 2023-05-03

**Authors:** Ya-Nan Yang, Zhixue Du, Wenhua Lu, Yue Qi, Yan-Qiang Zhang, Wan-Feng Zhang, Peng-Fei Zhang

**Affiliations:** ^1^ State Key Laboratory of Isotope Geochemistry, Guangzhou Institute of Geochemistry, Chinese Academy of Sciences, Guangzhou, China; ^2^ CAS Center for Excellence in Deep Earth Science, Guangzhou, China; ^3^ College of Earth and Planetary Sciences, University of Chinese Academy of Sciences, Beijing, China; ^4^ Faculty of Earth Resources, China University of Geosciences, Wuhan, China

**Keywords:** water, bridgmanite, NanoSIMS, high pressure, deep Earth

In the published article, there was an error in the **Funding** statement. One of the National Natural Science Foundation Grant Nos was displayed as “42021002.” The correct Grant No should be “42150102.” The correct **Funding** statement appears below.

“This work is supported by the National Natural Science Foundation of China (Grant Nos 42150102 and 41903017), the Strategic Priority Research Program (B) of the Chinese Academy of Sciences (Grant No. XDB18000000), Science and Technology Program of Guangzhou, China (202102020250), and the Director’s Fund of Guangzhou Institute of Geochemistry, CAS (2022SZJJZD-03 and 2020000173).”

The authors apologize for this error and state that this does not change the scientific conclusions of the article in any way. The original article has been updated.

