# Supplementary material for: NanoSIMS analysis of water content in bridgmanite at the micron scale: An experimental approach to probe water in Earth’s deep mantle
Source: Front Chem. 2023 Apr 7;11:1166593. doi: 10.3389/fchem.2023.1166593 (PMC10119403; doi:10.3389/fchem.2023.1166593)
Supplement: Supplementary file 1 [file DataSheet1.PDF]

Laser heated diamond anvil cell (LH-DAC)

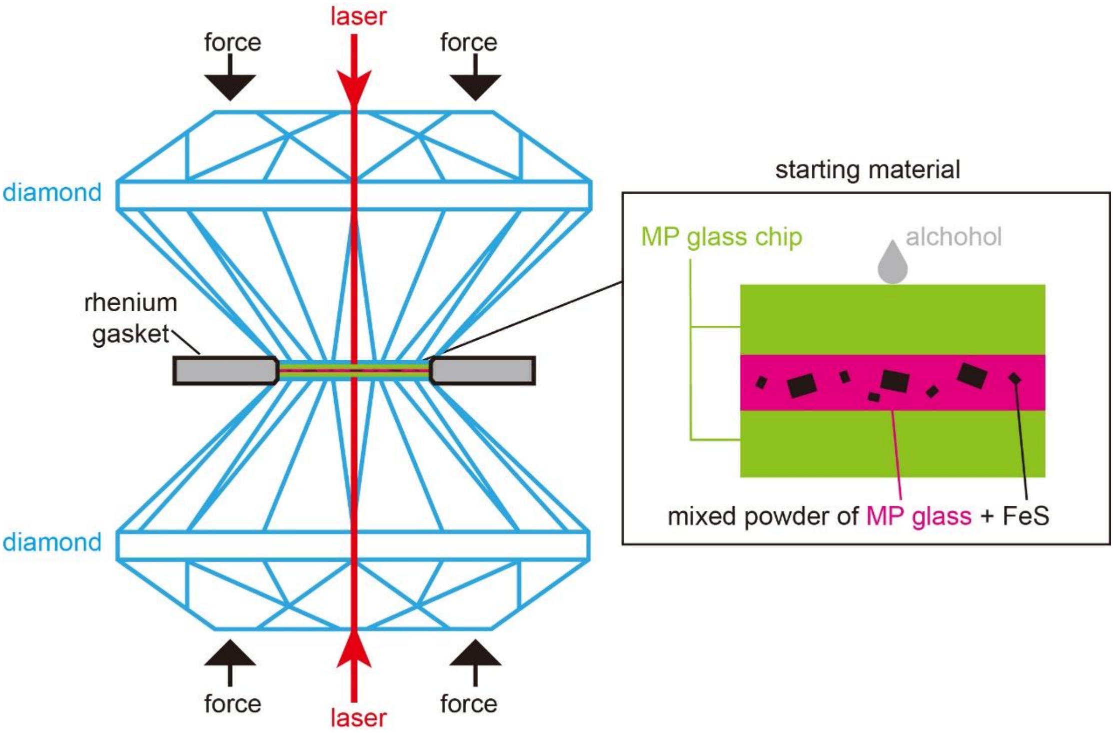

FIGURE S1. Schematic diagram showing LH-DAC with a close view of loaded starting materials. The mixed powder of F- and Cl-bearing mid-ocean basalt plus pyrolite (MP) glass and FeS is sandwiched between two pieces of MP glass chips. Alcohol is added to the surface MP glass on the top.

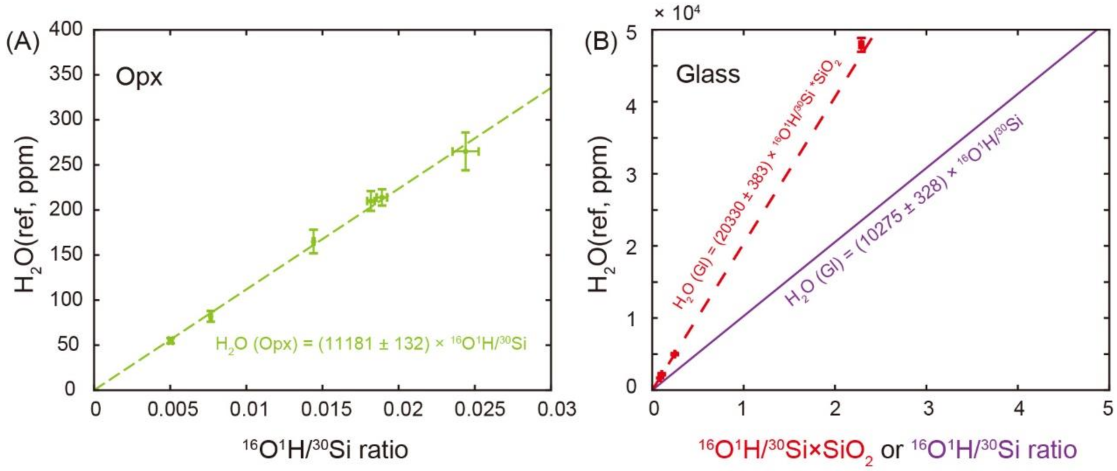

FIGURE S2. Calibration curves for bridgmanite and glass produced by LH-DAC by Opx (A) and glass reference materials (B). In B, two approaches to constructing the calibration curve for glass are displayed. It can be seen that using $^{16}O^{1}H/^{30}Si$ ratios that are not normalized by SiO_2 yields a slope that is about 8% lower than that of Opx reference materials.
